# Spatial distribution of tuberculosis incidence in Los Angeles County

**DOI:** 10.1186/s12889-020-09523-6

**Published:** 2020-09-21

**Authors:** Adam Readhead, Alicia H. Chang, Jo Kay Ghosh, Frank Sorvillo, Julie Higashi, Roger Detels

**Affiliations:** 1 Department of Epidemiology, Fielding School of Public Health, University of California, Los Angeles, Los Angeles, USA; 2 TB Control Program, Los Angeles County Department of Public Health, Los Angeles, USA; 3 Independent Researcher, Los Angeles, USA

## Abstract

**Background:**

In Los Angeles County, the tuberculosis (TB) disease incidence rate is seven times higher among non-U.S.-born persons than U.S.-born persons and varies by country of birth. But translating these findings into public health action requires more granular information, especially considering that Los Angeles County is more than 4000 mile^2^. Local public health authorities may benefit from data on which areas of the county are most affected, yet these data remain largely unreported in part because of limitations of sparse data. We aimed to describe the spatial distribution of TB disease incidence in Los Angeles County while addressing challenges arising from sparse data and accounting for known cofactors.

**Methods:**

Data on 5447 TB cases from Los Angeles County were combined with stratified population estimates available from the 2005–2011 Public Use Microdata Survey. TB disease incidence rates stratified by country of birth and Public Use Microdata Area were calculated and spatial smoothing was applied using a conditional autoregressive model. We used Bayesian Poisson models to investigate spatial patterns adjusting for age, sex, country of birth and years since initial arrival in the U.S.

**Results:**

There were notable differences in the crude and spatially-smoothed maps of TB disease rates for high-risk subgroups, namely persons born in Mexico, Vietnam or the Philippines. Spatially-smoothed maps showed areas of high incidence in downtown Los Angeles and surrounding areas for persons born in the Philippines or Vietnam. Areas of high incidence were more dispersed for persons born in Mexico. Adjusted models suggested that the spatial distribution of TB disease could not be fully explained using age, sex, country of birth and years since initial arrival.

**Conclusions:**

This study highlights areas of high TB incidence within Los Angeles County both for U.S.-born cases and for cases born in Mexico, Vietnam or the Philippines. It also highlights areas that had high incidence rates even when accounting for non-spatial error and country of birth, age, sex, and years since initial arrival in the U.S. Information on spatial distribution provided here complements other descriptions of local disease burden and may help focus ongoing efforts to scale up testing for TB infection and treatment among high-risk subgroups.

## Background

In the United States, TB disease incidence is notably higher among non-U.S.-born persons and incidence rates vary substantially by country of birth [1]. Los Angeles County has a diverse population of more than 10 million people, of which 3.5 million were born outside the U.S., and covers more than 4000 mile^2^ [2]. Substantial disparities in TB incidence by country of birth have been noted in Los Angeles County, however, the spatial distribution of TB incidence in Los Angeles County has largely remained unreported [3, 4].

We anticipate that TB disease is unevenly distributed given spatial analyses conducted in other locales [5, 6]. Information on areas of elevated TB disease are especially relevant now as scaling up targeted testing and treatment for TB infection has been shown to be an important component of TB elimination [7]. Only a fraction of those infected with TB will go on to develop TB disease. While sub-county data on TB infection prevalence is rarely available, TB incidence rates can function as a reasonable proxy. Estimating TB infection prevalence is difficult as it would require a large-scale effort to administer tests for latent TB infection to a random sample of the population. There has also been substantial interest in spatially targeted public health interventions [8, 9].

We used data from the Los Angeles County TB surveillance system and the American Community Survey to calculate crude TB incidence rates by country of birth and sub-county area. We then smoothed these maps using a spatial conditional autoregressive model to attenuate the effects of sparse data. Finally, we created extended models to account for additional covariates and non-spatial error.

## Methods

Data collection has been described previously [3]. Between 2005 and 2011, 5447 TB cases meeting the definition for the report of a verified case of tuberculosis (RVCT) were reported to the Los Angeles County Department of Public Health TB Control Program [10]. Addresses at diagnosis were geocoded using a Los Angeles Countywide Address Management System locator and spatially joined to allow the case to be assigned to one of 67 Public Use Microdata Areas (PUMAs) in Los Angeles County as defined by the 2000 U.S. Census [11]. This study was deemed exempt by the Los Angeles County Department of Public Health Institutional Review Board. Data for population estimates stratified by PUMA and other covariates of interest were obtained from the Integrated Public Use Microdata Series, a curated copy of the U.S. Census’s Public Use Microdata Survey and other microdata [12]. PUMAs were chosen as the geography of interest because they were the smallest area for which the full joint distribution of key covariates was available. These covariates were age at diagnosis, sex, country of birth and years since initial arrival in the U. S, which was defined as years since the year of immigration. Population estimates were calculated using replicate weights; exclusions, replicate weights, and sampling frame were discussed in detail in prior work [3]. After excluding 494 (9%) cases due to missing data or differences in sampling frame between Los Angeles County TB surveillance and the American Community Survey, 4953 cases were available for analysis. Years since initial arrival in the U.S. was found to be strongly associated with TB incidence in previous studies [1]. To accommodate the inclusion of years since initial arrival in multivariable models, the data were further limited to non-U.S.-born cases, leaving 3945 cases for analysis. Cases residing in the cities of Long Beach or Pasadena were not included as those cases are not reported to the Los Angeles County Department of Public Health.

Crude TB incidence rates stratified by PUMA alone and by country of birth and PUMA were calculated. Spatial smoothing was achieved using Bayesian Poisson model with a conditional autoregressive term from Besag et al. which is used frequently in spatial applications [13]. The preliminary model (Eq. 1 below) accounts for area and country of birth only. Following the notation of Kleinschmidt et al., *Y*_*ic*_ is defined as the observed diagnoses occurring in area *ⅈ* and among country of birth *c*; *P*_*ic*_ is defined as the person-time for the same stratum [14]. Additionally, we define *η*_*ic*_ ≡ *E*[*Y*_*ic*_] and assume that *Y*_*ic*_~*Poisson*(*η*_*ic*_). The transformed linear regression is then:

1 $$ \mathit{\log}\left({\eta}_{ic}\right)=\log \left({P}_{ic}\right)+\alpha +{\beta}_c{X}_c+{\varphi}_i $$

where *φ*_*i*_ denotes a spatially-correlated random effects term defined by the following [13]:

$$ {\varphi}_i\mid {\varphi}_{-i}=N\left(\overline{\varphi_i},\frac{{\sigma_{\varphi}}^2}{n_i}\ \right) $$

$$ \overline{\varphi_i}=\frac{1}{n_i}\sum \limits_{j\in neighbors\ of\ i\ }{\varphi}_{i.} $$

Neighbors of area *ⅈ* were defined with queen-style contiguity.

Subsequent models (eqs. 2 and 3), adjusted for covariates age at diagnosis, sex, country of birth, and years since initial arrival, used the following transformed linear regressions:

2 $$ \mathit{\log}\left({\eta}_{is}\right)=\log \left({P}_{is}\right)+\alpha +\boldsymbol{\beta} \boldsymbol{X}+{\varphi}_i $$

3 $$ \mathit{\log}\left({\eta}_{is}\right)=\log \left({P}_{is}\right)+\alpha +\boldsymbol{\beta} \boldsymbol{X}+{\varphi}_i+{\omega}_s $$

where *s* denotes the stratum, ***βX*** denotes the vectors of covariates and covariate betas, *ω*_*s*_ denotes the spatially-uncorrelated heterogeneity with the distribution *ω*_*s*_~*N*(0, *σ*^2^). The priors were set as follows: *α* was given a flat prior, ***β*** were given N (0, 1000), and *φ*_*i*_ and *ω*_*s*_ were both given Gamma (0.5, 2000). Bayesian models were run with two chains for 100,000 iterations and 10,000 iterations of burn-in. Mixing was evaluated through visual inspection of caterpillar plots and density charts. ArcGIS 10.0 was used to geocode and assign PUMA geography. R version 3.4, R Studio version 1.0.143 and a variety of packages were used to manage and analyze data and create maps [15–21]. Bayesian models were run in OpenBUGS version 3.2.2 rev 1012 [22]. Due to limitations stemming from sparse data for most country-of-birth groups, only a select group of countries of birth were analyzed via crude and spatially-smoothed TB incidence. Data from all country-of-birth groups were included in subsequent adjusted models.

## Results

**Fig. 1:**
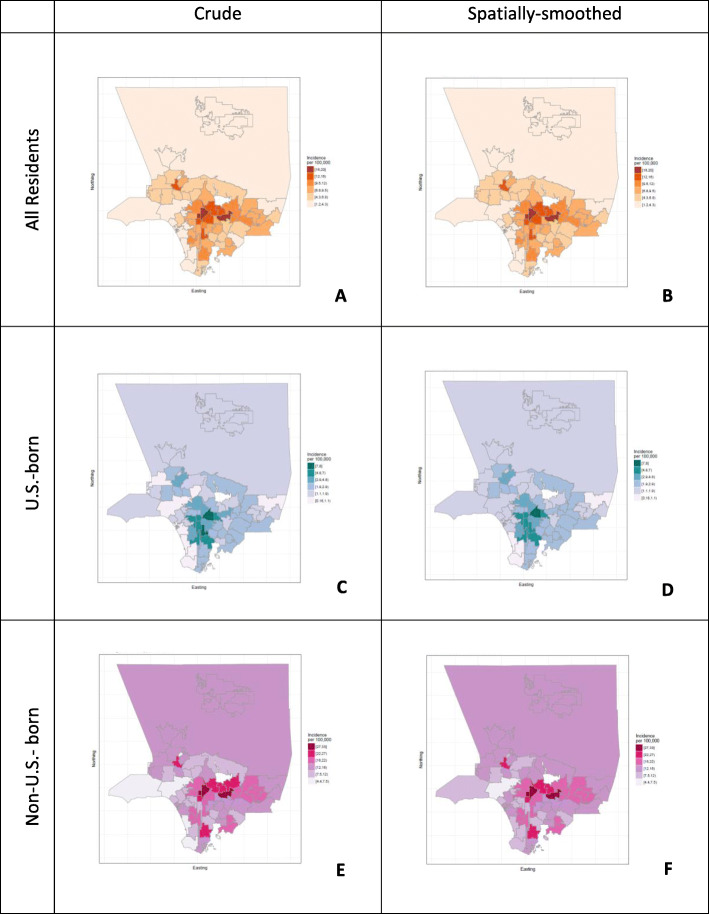
Crude and Spatially-smoothed TB Incidence per 100,000 among Selected Subgroups, Los Angeles County 2005–2011. Note: Estimates of TB incidence for Pasadena and Long Beach were not calculated as data for these cases are not reported to the Los Angeles County Department of Public Health. Map created with R under the GNU library general license version 2

The tuberculosis average annual incidence in Los Angeles County 2005–2011 was 7.2 per 100,000 person-years; the rate among U.S.-born persons was 2.3 per 100,000 in contrast to the rate among non-US-born persons which was occurring among 15.8 per 100,000 [3]. The map for crude incidence among all residents shows higher incidence in central areas of the county and lower incidence in outer areas (Fig. 1a). For reference, the California and U.S. TB disease incidence rate in the same period were 7.1 per 100,000 and 4.1 per 100,000 respectively [23]. Areas of notable high incidence include Panorama City, Pico Heights and Echo Park, and Monterey Park-Rosemead, which are in the northwest, center, and east sections of the county, respectively. These areas had crude incidence rates of 13.2, 19.7, 17.2 and 19.2 TB cases per 100,000 respectively. Spatial smoothing had minimal effect on estimates (Fig. 1b); median absolute difference between spatially-smoothed and crude incidence rates was 0.13 per 100,000 with a maximum of 0.59 per 100,000.

TB incidence among U.S.-born persons and non-U.S.-born persons showed different spatial patterns. Among U.S.-born persons, there were areas of high incidence in Los Angeles City downtown, Watts, and East Los Angeles (Fig. 1c, d**)**. These areas had incidence rates of 8.0, 7.9 and 6.1 per 100,000 respectively. In contrast, among non-U.S.-born persons, TB incidence was notably higher in Monterey Park/Rosemead, Pico Heights, and Echo Park. These areas had TB incidence rates of 32.8, 26.2 and 28.3 per 100,000 respectively. Also noteworthy were two areas of elevated incidence that are not contiguous with the area of elevated incidence at the center of the county, specifically Panorama City (northwest) that had an incidence rate of 23.1 per 100,000 and Carson (about 18 miles south of downtown) that had an incidence rate of 25.1 per 100,000. Changes in estimates via spatial smoothing for both U.S.-born and non-U.S.-born persons were minor (Fig. 1d, f).

**Fig. 2:**
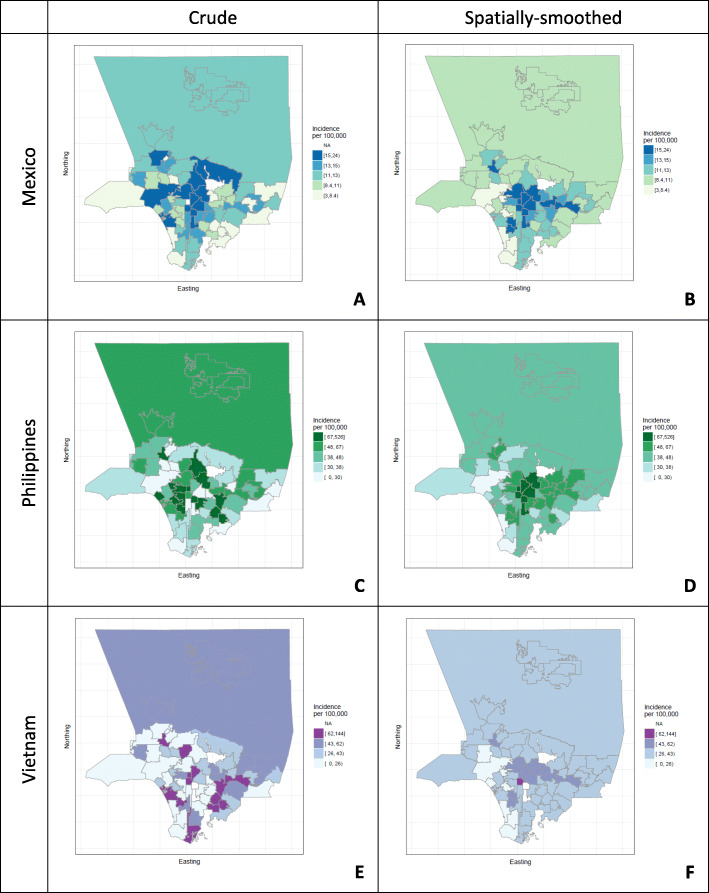
Crude and Spatially-smoothed TB Incidence among Selected Countries of Birth, Los Angeles County 2005–2011. Note: Estimates of TB incidence for Pasadena and Long Beach were not calculated as data for these cases are not reported to the Los Angeles County Department of Public Health. Map created with R under the GNU library general license version 2

Prior reports have shown notable differences in incidence by country of birth with the largest absolute number of cases occurring among persons born in Mexico, Philippines or Vietnam [3, 4]. The map for crude incidence rates among persons born in Mexico shows a condensed spatial form centered north of downtown Los Angeles in contrast to maps for crude incidence among persons born in the Philippines or Vietnam which show more dispersed patterning throughout the county (Fig. 2a, c, e). Maps of spatially-smoothed incidence rates had a less dispersed pattern than crude maps and show concentrated areas of high incidence in the center of the county (Fig. 2b, d, and f). Maps for spatially-smoothed incidence rates among persons born in Mexico or the Philippines show a cluster of areas of high incidence centered on the Los Angeles City downtown (Fig. 2b, d). The spatially-smoothed map for incidence rates among persons born in Vietnam shows a small area of high incidence centered on the Los Angeles downtown (Fig. 2f).

**Fig. 3:**
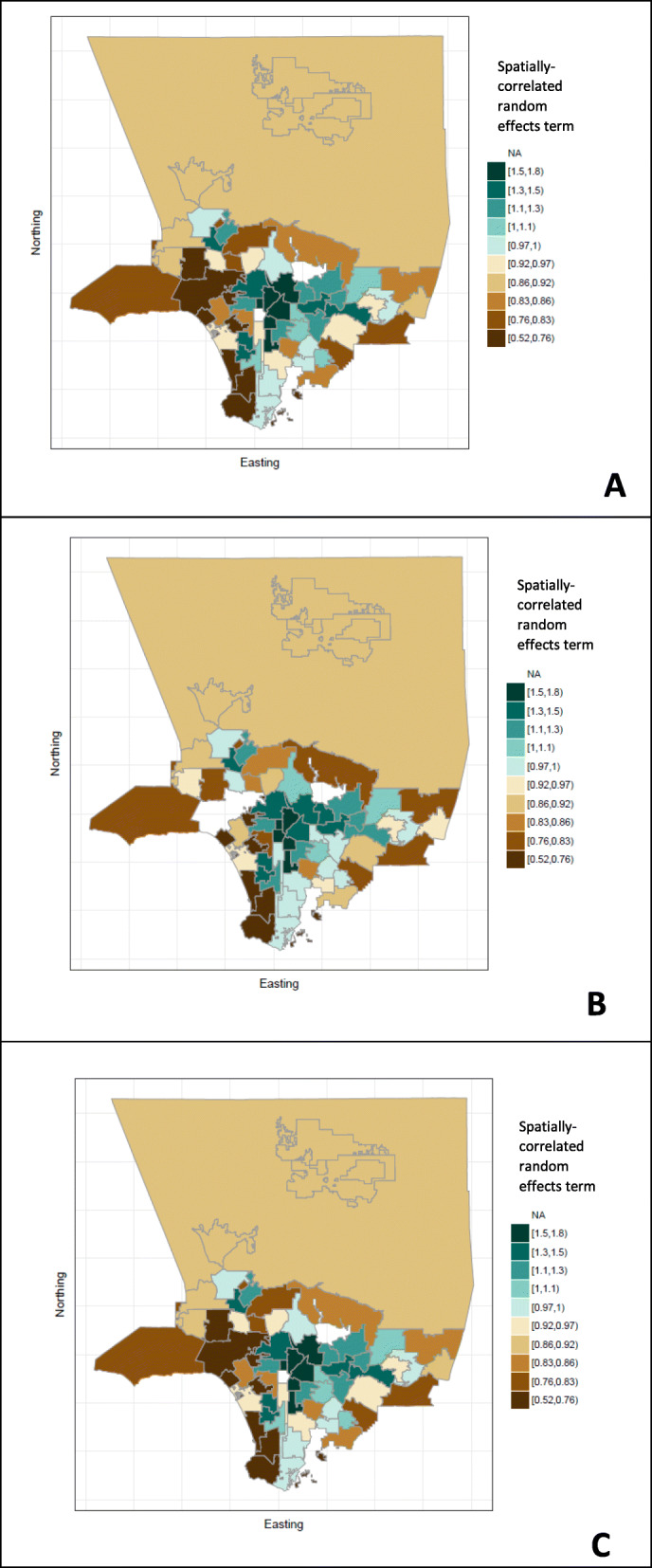
Three Bayesian models of TB Incidence Spatial Component, Los Angeles County 2005–2011. Note: The first model was adjusted for country of birth only (**a**). The second model was adjusted for country of birth, age, sex and years since initial arrival (**b**). The third model was adjusted for country of birth, age, sex, years since initial arrival and non-spatial error (**c**). Estimates of TB incidence for Pasadena and Long Beach were not calculated as data for these cases are not reported to the Los Angeles County Department of Public Health. Map created with R under the GNU library general license version 2

Subsequent adjusted models which included age, sex, country of birth and years since initial arrival showed condensed spatial patterns (Fig. 3). Maps show spatial clustering even when accounting for these covariates and non-spatially correlated error.

## Discussion

TB disease incidence rates were uneven across Los Angeles County, both for TB cases overall and for country-of-birth subgroups that were analyzed. Areas of high incidence among U.S.-born persons were evident in downtown Los Angeles as well as to the east of the city center. Among persons born the Philippines or Vietnam, crude TB incidence rates exhibited a highly-dispersed spatial pattern. In contrast, among persons born in Mexico, a condensed spatial pattern in crude TB incidence was evident. Maps of spatially-smoothed TB incidence rates showed areas of high incidence centered on the Los Angeles downtown area. For the local clinical community, we believe that this information can add supportive detail to a clinical risk assessment. The California Department of Public Health TB Control Branch recently issued a tuberculosis risk assessment that underscores the importance of country of birth in determining TB risk [24]. Additional detail on country of birth specific risks and risks specific to local community areas could also help providers.

The notable differences between the crude and smoothed maps for the selected countries of birth show the utility of spatial smoothing. In the crude maps, low absolute values of strata numerators and denominators for persons born in the Philippines or Vietnam produced highly variable incidence estimates. The smoothed maps are easier to interpret because high variability of areas with sparse data has been attenuated. These maps would be the preferred starting point in assessing burden and identifying areas where “place-based” interventions could be focused.

Spatial patterning persisted even when adjusting for country of birth, age, sex, years since initial arrival and non-spatial error. The notable spatial heterogeneity as evidenced by the spatially-correlated random effects term for the country of birth only model suggests that models with additional covariates were justified to explain spatial differences, in that country of birth alone was not sufficient to explain the existing spatial pattern (Fig. 3a). However, two further models, one using additional covariates and another with additional covariates and non-spatial error, attenuated but did not remove this spatial heterogeneity (Fig. 3b and c). This suggests that these models cannot fully explain the spatial distribution of TB incidence. Additional data, such as data on recent transmission and socio-economic status, may improve future models of the spatial component of TB disease [25, 26].

This analysis has additional limitations beyond issues of case ascertainment and survey error discussed in prior work [3]. This analysis is vulnerable to the modifiable areal unit problem (MAUP) and may yield different results based on the size and shape of the areas under study. Low absolute numbers in strata numerators and denominators make incidence calculations more variable. Areas on the edge of the county have fewer neighbors and so may not be as well smoothed as those in the middle of the county. PUMA boundary definitions from the 2000 Census allowed for non-contiguous areas, which could have distorted the smoothing process by creating neighbors for non-contiguous areas. Also, cases from Long Beach and Pasadena are not included here, as they belong to public health departments distinct from Los Angeles County. As a result, areas around Pasadena and Long Beach are missing a neighbor area and so are not smoothed as they would be if those cases had been included.

## Conclusion

TB disease incidence is spatially heterogeneous within Los Angeles County and remained so when stratified by country of birth and after accounting for age, sex, years since initial arrival and non-spatial error. The spatial patterning in the maps provides complementary information to descriptions of the local disease burden. This information informs public health planning by identifying areas of high incidence where interventions can be focused. For example, public health outreach focused on these high incidence areas could take the form of local education activities for the public and health care providers on TB targeted testing and new treatment regimens to prevent TB reactivation. These analyses could be further extended by using ecological variables, such as crowding or other socio-economic indicators, and by separately analyzing cases identified as resulting from recent transmission [26].

This study reinforces the importance of spatial data in local description of TB epidemiology and suggests their utility in enhancing predictive models of TB incidence.

## Data Availability

The datasets analyzed during the current study are not publicly available as they contain sensitive personal health information that is protected by the federal HIPAA Privacy Rule and cannot be publicly shared. Application for access should be addressed to the Los Angeles County Department of Public Health TB Control Program.

## Abbreviations

TB: Tuberculosis
PUMA: Public use microdata area
